# Continuous Genomic Surveillance Monitored the *In Vivo* Evolutionary Trajectories of Vibrio parahaemolyticus and Identified a New Virulent Genotype

**DOI:** 10.1128/mSystems.01254-20

**Published:** 2021-01-19

**Authors:** Songzhe Fu, Qian Yang, Qingyao Wang, Bo Pang, Ruiting Lan, Dawei Wei, Baocheng Qu, Ying Liu

**Affiliations:** a College of Marine Science and Environment, Dalian Ocean University, Dalian, China; b Key Laboratory of Environment Controlled Aquaculture (KLECA), Ministry of Education, Dalian, China; c Center for Microbial Ecology and Technology (CMET), Ghent University, Ghent, Belgium; d State Key Laboratory of Infectious Disease Prevention and Control, National Institute for Communicable Disease Control and Prevention, Chinese Center for Disease Control and Prevention, Beijing, China; e School of Biotechnology and Biomolecular Sciences, University of New South Wales (UNSW), Sydney, NSW, Australia; f Institute of Microbiology, Chinese Academy of Sciences, Beijing, China; Cleveland Clinic

**Keywords:** acute hepatopancreatic necrosis disease (AHPND), *Vibrio parahaemolyticus*, mobile genetic elements (MGEs), evolutionary trajectories, IS*Val1*

## Abstract

Our ability to predict evolutionary trajectories of pathogens is one of the promising leverages to fight against the pandemic disease, yet few studies have addressed this question *in situ*, due to the difficulty in monitoring the milestone evolutionary events for a given pathogen and in understanding the evolutionary strategies. In this study, we monitored the real-time evolution of Vibrio parahaemolyticus in response to successive antibiotic treatment in three shrimp farms in North China from 2011 to 2018 by whole-genome sequencing. Results showed that the stepwise emergence of resistance was associated with the antibiotic usage. Genomic analysis of resistant isolates showed that the acquisition of the resistant mobile genetic elements flanked by an insertion sequence (IS*Val1*) closely mirrored the antibiotics used in shrimp farms since 2014. Next, we also identified 50 insertion sites of IS*Val1* in the chromosome, which facilitated the formation of pathogenicity islands (PAIs) and fitness islands in the following years. Further, horizontal transfers of a virulent *trh*-*nik-ure* genomic island (GI) and two GIs improving the fitness have been observed in two farms since 2016. In this case study, we proposed that the insertion sequence triggered four major evolutionary events during the outbreaks of shrimp disease in three farms, including horizontal transfer of transposon (HTT) (stage 1), the formation of resistance islands (stage 2) and the PAIs (stage 3), and horizontal transfer of the PAIs (stage 4). This study presented the first *in vivo* evolutionary trajectories for a given bacterial pathogen, which helps us to understand the emergence mechanisms of new genotypes.

**IMPORTANCE** Most human infectious diseases originate from animals. Thus, how to reduce or prevent pandemic zoonoses before they emerge in people is becoming a critical issue. Continuous genomic surveillance of the evolutionary trajectories of potential human pathogens on farms is a promising strategy to realize early warning. Here, we conducted an 8-year surveillance of Vibrio parahaemolyticus in three shrimp farms. The results showed that the use of antibiotics and horizontal transfer of transposons (HTT) drove the evolution of V. parahaemolyticus, which could be divided into four stages: HTT, formation of resistance islands, formation of pathogenicity islands (PAIs), and horizontal transfer of PAIs. This study presented the first *in vivo* monitoring of evolutionary trajectories for a given bacterial pathogen, providing valuable information for the prevention of pandemic zoonoses.

## Abstract (precis)

Most human infectious diseases originate from animals. Thus, how to reduce or prevent pandemic zoonoses before they emerge in people is becoming a critical issue.

## INTRODUCTION

Most human infectious diseases, especially recently emerging pathogens, originate from animals (1), and ongoing transmission of diseases such as coronavirus disease 2019 (COVID-19) presents a significant global health crisis (2). The recent emergence of severe acute respiratory syndrome (SARS), Middle East respiratory syndrome (MERS), and COVID-19 has shown the vulnerability of humans to novel zoonotic pathogens and highlights that pathogen transmission from animals to humans can cause devastating consequences (3).

Although prediction of the emergence of novel pandemic agents often seems unachievable, efforts to reduce or prevent pandemic zoonoses before they emerge in people have also begun (4). For instance, Daszak et al. developed a model dividing cross-species transmission into three stages, animal infection without transmission to humans (stage 1), localized human infection (spillover; stage 2), and widespread human-to-human transmission (pandemic; stage 3) (5). Recent recognition that most emerging infectious disease events have wildlife origins highlights the need for a deep understanding of the evolutionary drivers for pathogens in stages 1 and 2 and the type of contact between animals and people that enables disease transmission (6). Opportunities for close contact between humans and wild animals are relatively rare compared to contact with domestic animals (7). Therefore, our ability to monitor the emergence and spread of pandemic diseases from farms to humans is critical to mitigate the exacerbation of the infectious disease crisis worldwide.

Continuous surveillance is essential for recognition of the evolutionary events involved in the cross-species transmission, spillover, and the spread of diseases, which can be used to predict the future disease emergence risks (8). The emergence of a pandemic pathogen transmitted from animals to humans usually involves a series of evolutionary events (9). A genomics-based pathogen surveillance system enables the real-time surveillance of such evolution (10).

For instance, a large outbreak of infections was reported in Germany in 2011, which was caused by Shiga toxin-producing Escherichia coli serotype O104:H4 (11). Genomic analysis revealed that this exceptionally virulent serotype acquired virulence factors from enteroaggregative E. coli, conferring mixed enteroaggregative and Shiga toxin-producing abilities and resulting in the emergence of a new virulent serotype (12).

*In vivo* monitoring of such evolutionary events becomes a long-standing issue. For example, Vibrio parahaemolyticus is a Gram-negative waterborne pathogen which has a large chromosome (chromosome 1) and a small chromosome (chromosome 2) (13). Recently, V. parahaemolyticus has become the leading cause of seafood-borne gastroenteritis (14). A recent phylogenetic analysis of pandemic V. parahaemolyticus isolates (emerged in 1996), and prepandemic isolates (isolated in the 1980s) suggested that the prepandemic strains initially acquired a genomic island containing thermostable direct hemolysin gene (*tdh*) and a *toxRS* new region (15); subsequent genomic analysis revealed that acquisition of seven novel genomic islands converted them into pandemic O3:K6 isolates within a decade (16).

Thus, such evolutionary events should be carefully monitored because once a pathogen has spilled over from farm animals to humans, human-to-human transmission of zoonoses can occur rapidly. However, few studies have addressed this issue *in situ* from farms to humans due to the difficulty of real-time surveillance of a given pathogen’s evolution and its precise microevolution in the long term. The recent next-generation sequencing revolution provided an unprecedented opportunity to explore the emergence of pandemic pathogens *in situ* (17). Whole-genome sequencing (WGS) has yielded novel insights into pathogen evolution, allowing inferences regarding population dynamics and geographic spread at both local and global levels (18). Acquisition of resistance by a given strain through horizontal transfer of mobile elements can also be monitored using this methodology. For instance, Du et al. sampled isolates during a 3-year large-scale outbreak of furunculosis in an Atlantic salmon farm and reconstructed the precise timeline of resistance island acquisition events in response to antibiotic pressure (19).

However, very little is currently known about the time needed for a given pathogen to transit from stage 1 to stage 2; that is, no *in situ* data are available to monitor the time required for an infectious agent in animals to become contagious to humans. In particular, the details of the microevolution and emergence of a pathogen from farms to humans remain largely unknown, as no long-term surveillance data from farms are available.

Consumption of undercooked seafood is one of the most essential infection routes for pathogenic V. parahaemolyticus-related infection (13). A recent study suggested that V. parahaemolyticus can also cause acute hepatopancreatic necrosis disease (AHPND) in shrimp due to the acquisition of a 70-kb plasmid encoding the binary toxin PirAB^vp^ (20). The *pirAB*^vp^ gene was flanked by two identical insertion sequences named IS*Val1* and form a mobile genetic element (MGE) called Tn*6264* (21). Our previous studies suggested that IS*Val1* can excise from the plasmid and insert into the chromosomes both *in vivo* and *in vitro* (22). AHPND was first identified in South China and Vietnam in 2010 (20) and then reported in East China in 2011 and North China in 2013, which provided an extraordinary opportunity to observe the *in vivo* evolution of V. parahaemolyticus during the spread of this zoonosis. To record the spread of AHPND in North China, we monitored the real-time evolution of V. parahaemolyticus in three nearby shrimp-farming regions from 2011 to 2018 and accidentally found that IS*Val1* mediated the evolution of V. parahaemolyticus.

## RESULTS

### Progression of shrimp disease and the development of antibiotic resistance of V. parahaemolyticus in Tianjin.

We conducted continuous pathogen monitoring in three nearby shrimp farms in Tianjin, Tangshan, and Huanghua cities, China, to observe the microevolution of V. parahaemolyticus *in vivo* (Fig. S1). In Tianjin, shrimps were cultured in a recirculating aquaculture system (RAS) which consisted of ponds and a wastewater treatment unit, whereas shrimps were cultivated in open ponds in Tangshan and Huanghua without wastewater treatment units. From 2011 to 2018, diseased shrimps were sampled from each outbreak-related pond annually to isolate the V. parahaemolyticus. The V. parahaemolyticus isolates were then subjected to multilocus sequence typing (MLST) to confirm the sequence type (ST) (23). The presence of virulence factors, including *pirAB^vp^*, T3SS1, T3SS2, and *tdh/trh*, were also screened by PCR (24–26).

10.1128/mSystems.01254-20.1 FIG S1 The location of sampled shrimp farms in Tianjin, Tangshan and Huanghua. The sampling sites were mapped by the ArcGIS Desktop 10.2 software. Download FIG S1, PDF file, 1.0 MB. Copyright © 2021 Fu et al. 2021 Fu et al. This content is distributed under the terms of the Creative Commons Attribution 4.0 International license.

**FIG 1:**
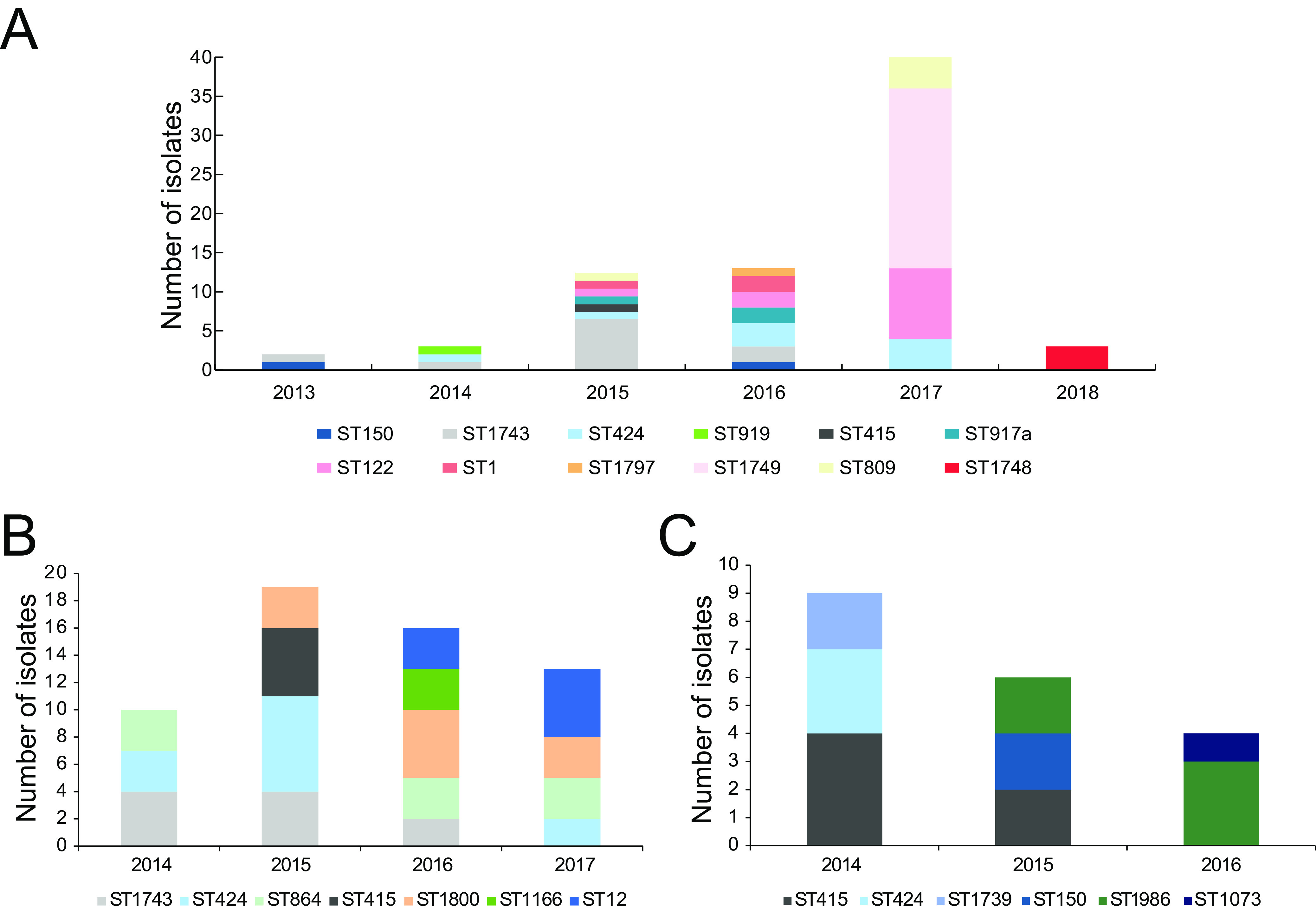
(A to C) The dynamics of V. parahaemolyticus sequence types (STs) from 2011 to 2018 obtained from three-shrimp farming regions in Tianjin (A), Tangshan (B), and Huanghua (C), China.

From 2011 to 2012, no V. parahaemolyticus was isolated and no massive shrimp disease was recorded in three farming regions. In 2013, an occasional outbreak of shrimp disease was first identified in Tianjin; oxytetracycline was only occasionally used for treatment. Only one ST1743 strain and one ST150 strain were identified in the shrimps in 2013, of which ST150 strain VP17 was a *pirAB^vp^*-positive strain (Table S1). This is the first record of a *pirAB^vp^*-positive strain on this farm. Accordingly, except for the resistance to amoxicillin, no antibiotic resistance out of nine tested antibiotics was found in 2013 isolates (Table S2). In July 2014, the outbreak was identified in 30% of the ponds. Three V. parahaemolyticus strains were isolated and were divided into ST424, ST1743, and ST919, respectively (Fig. 1A). All of them were *pirAB^vp^*-positive. As the outbreak spread to more ponds, oxytetracycline and florfenicol were then widely used to manage the outbreak beginning in 2014. Meanwhile, to eliminate the introduced V. parahaemolyticus, the seawater used for evaporation compensation was treated with ozone for 30 min before it entered the pond.

10.1128/mSystems.01254-20.4 TABLE S1 Virulence and plasmid profiles of genomes used in this study. Download Table S1, XLSX file, 0.02 MB. Copyright © 2021 Fu et al. 2021 Fu et al. This content is distributed under the terms of the Creative Commons Attribution 4.0 International license.

10.1128/mSystems.01254-20.5 TABLE S2 Antibiotic resistance profile of sequence strains in three shrimp farms. Download Table S2, XLSX file, 0.02 MB. Copyright © 2021 Fu et al. 2021 Fu et al. This content is distributed under the terms of the Creative Commons Attribution 4.0 International license.

However, the combined use of antibiotics did not stop the outbreaks, which affected 50% of the ponds in July 2015. Fifteen strains were isolated from the shrimp ponds, which were subtyped into ST1, ST1743, ST424, ST415, ST809, ST1749, ST122, and ST917a. Resistance to florfenicol was detected in ST1, ST122, and ST809 strains in 2015.

Due to the emergence of florfenicol-resistant strains in 2015, antibiotic treatment was shifted to sulfamethoxazole, enrofloxacin, and streptomycin. However, the situation still became worse in July 2016 as outbreaks spread to more than 80% of the ponds; 14 V. parahaemolyticus strains were identified in the shrimp, including 4 *pirAB^vp^*-positive STs (ST1743, ST424, ST150, ST917a) and three *pirAB^vp^*-negative STs (ST1, ST122, and ST1797). Resistance to amoxicillin, sulfamethoxazole, streptomycin, and florfenicol was identified in ST1797, ST1, and ST122 strains. In April 2017, a larger outbreak occurred and spread out to almost all ponds, resulting in the isolation of 39 V. parahaemolyticus strains that can be subtyped into ST1, ST122, ST809, ST1749, and ST424. Corresponding multiple-drug resistances were found in ST1, ST122, and ST809 strains. For the virulence genes, T3SS1 was found in all isolates, while T3SS2 was only presented in ST1 isolates which were *trh*-positive. Interestingly, *trh* was also amplified in one ST424 strain by PCR.

Due to the severe outbreaks in shrimp culture, all of the shrimp ponds were cleaned up and replaced with fresh rearing water in July 2018. Microbiological analysis of 60 shrimp samples from 4 ponds only found three ST1748 isolates.

### Progression of shrimp disease in Tangshan and Huanghua.

Just 1 year after AHPND was identified in Tianjin, suspected symptoms of AHPND were also identified in shrimp farms from Tangshan and Huanghua in 2014. Since then, V. parahaemolyticus has been continuously isolated from shrimp. Initially, the majority of them belonged to ST424, ST1743, and ST415 during 2014 to 2015 (Fig. 1B and C). Since 2015, several other genotypes have also been identified, including ST150, ST864, ST1800, ST1166, ST1073, and ST12. Interestingly, Tianjin, Tangshan, and Huanghua shared similar genotypes of V. parahaemolyticus (including ST424, ST1743, ST150, and ST415) from 2013 to 2015, probably because the close locations facilitate the exchange of V. parahaemolyticus. Apart from T3SS1, virulence genes were not found in the Tangshan and Huanghua data sets.

As the outbreak spread to more ponds since 2014, oxytetracycline and florfenicol were both used in the Tangshan and Huanghua ponds. Moreover, combined usage of sulfamethoxazole, streptomycin, and florfenicol was also applied to the ponds in 2016. However, all treatments failed to control the outbreaks. Farming activities ceased in Huanghua and Tangshan in 2016 and 2017, respectively.

### Antibiotic resistance was conferred by mobile gene elements (MGEs) with IS.

The above-described observation provides a clear example to show the emergence of resistance linked to the use of antibiotics. These observations raised the speculation that whether the stepwise acquisition of MGEs was mediated by IS*Val1*, which was previously found to be involved with the formation of resistance islands (22). Next, we conducted PCR to detect the presence of IS*Val1* in the isolates collected from three farms, 26 of which were IS*Val1*-positive. To understand whether IS*Val1* shaped the genomic plasticity as found previously (22), all IS*Val1*-positive isolates and the isolates from other representative STs in each year were selected for WGS.

**FIG 2:**
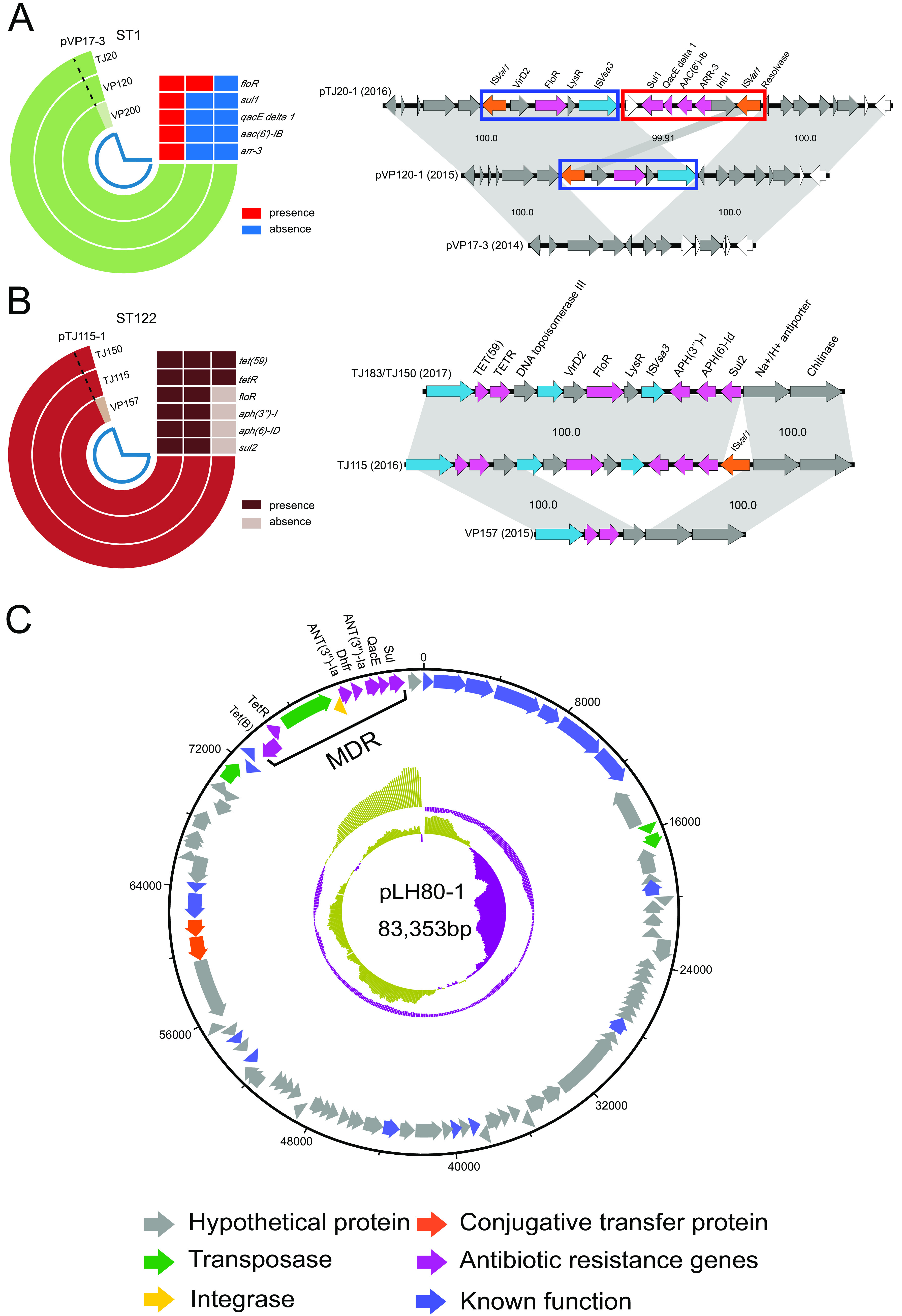
(A to C) Schematic diagrams showing the comparative genome analysis of ST1 (A), ST122 (B), and plasmid pLH80-1 (C) and the stepwise formation of resistant MGEs; the maximum-likelihood tree of V. parahaemolyticus is vertically on the top of rings; the antibiotic resistance genes of each isolates are shown under the tree.

Except for the intrinsic resistance to beta-lactam, no antibiotic resistance genes (ARGs) were found in 2013 and 2014 isolates. A genomic analysis of the isolates from 2015 showed that the emergence of resistance to florfenicol coincided with the acquisition of a plasmid harboring *floR* and IS*Val1* in an ST1 isolate, VP120 (Fig. 2A). This plasmid was also found in 2013 strain VP17 but without IS*Val1*. In 2016, the plasmid in an ST1 strain further acquired an additional composite transposon flanked by IS*Val1*, which contains resistance genes *sul1*, *aac(6′)-Ib-cr*, and ARR-3. Accordingly, multiple-drug resistance emerged in an ST1 strain in 2016 (Table S2).

In addition, a stepwise acquisition of ARGs was also found in ST122 isolates. In 2015, ST122 isolate VP157 only harbored four ARGs (*blaCARB-17*, *blaCARB-20*, *sul2*, and *tet* [59]). In 2016, one ST122 isolate acquired another resistance island (flanked by IS*Val1*) containing *floR*, *sul2*, *aph(6)-Id*, and *aph(3′')-Ib* (Fig. 2B). In 2017, the above-described ARGs was still present in one ST122 and one ST809 isolate but without IS*Val1.* Additionally, one ST1797 isolate harbored *qnrS5* in 2016, suggesting the role of IS*Val1* in the response to the antibiotic treatment and the acquisition of ARGs (Table S2).

Likewise, acquisition of resistance islands was also observed in the Tangshan and Huanghua data sets. ST1800 strains did not harbor any transferable ARGs in 2015. However, a resistance island conferring resistance to tetracycline was identified in a plasmid of ST1800 isolates in 2016 (Fig. 2C). Additionally, another four ARGs [*aph (3′')-Ia*, *sul1*, *qacE*, and *dfrA27*] were also identified in this plasmid. Moreover, the ST150 isolate HB23 also harbored this plasmid in 2017.

### Genomic analysis of insertion sites of IS*Val1* in the chromosomes of V. parahaemolyticus.

The discovery of resistant MGEs with IS*Val1* in V. parahaemolyticus prompted us to make a thorough investigation of the insertion sites of IS*Val1* in the chromosome of V. parahaemolyticus from three farms.

**FIG 3:**
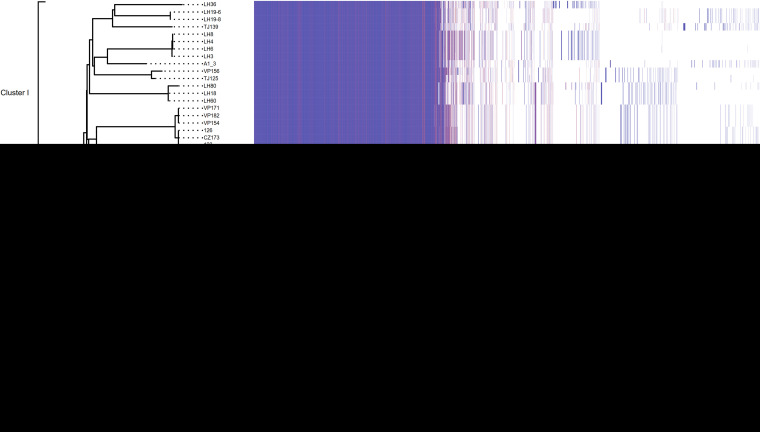
Maximum-likelihood (ML) phylogeny and the presence/absence variation (PAV) matrix in the 60 V. parahaemolyticus genomes collected in three shrimp farms; the scale bar denotes substitutions per variable site; the PAV matrix per isolate is ordered alongside the phylogenetic tree. The presence of the gene family is indicated by a block per column (the colors range from red to blue).

Genomic analysis of 60 sequenced genomes divided them into five clusters (Fig. 3) and identified 61 insertion events in the ST1, ST122, ST150, ST424, ST1166, ST1743, and ST917a isolates, which were scattered around the two chromosomes (Table S3).

10.1128/mSystems.01254-20.6 TABLE S3 Insertion sites of IS*Val1* in V. parahaemolyticus isolates from three farms. Download Table S3, XLSX file, 0.02 MB. Copyright © 2021 Fu et al. 2021 Fu et al. This content is distributed under the terms of the Creative Commons Attribution 4.0 International license.

In the Tianjin data set, 2013 strain VP17 has five IS*Val1* insertions in the chromosomes (Table S3), two of which resulted in the truncation of genes encoding aminotransferase class V-fold pyridoxal 5′-phosphate (PLP)-dependent enzyme and hypothetical protein (Fig. S2). Another ST1743 strain in 2013 was negative for both *pirAB^vp^* and IS*Val1*. In 2014, IS*Val1* was found in the chromosome of a ST424 isolate VP131, which inserted between two hypothetical proteins (Table S3). In 2015, apart from two ST1 strains with resistant MGEs flanked by IS*Val1*, six isolates were also IS*Val1*-positive in their chromosomes, of which ST424 strain 20151007009 had three genes disrupted by IS*Val1* (Fig. S2). This suggests that IS*Val1* has actively inserted into more genomes during the spread of AHPND. In July 2016, IS*Val1* was found in the chromosome of ST424, ST1, and three ST917a isolates.

10.1128/mSystems.01254-20.2 FIG S2 The disruption of functional genes by IS*Val1* in ST150 and ST424 strains. Download FIG S2, PDF file, 0.2 MB. Copyright © 2021 Fu et al. 2021 Fu et al. This content is distributed under the terms of the Creative Commons Attribution 4.0 International license.

In the Tangshan data set, only one ST424 isolate harbored IS*Val1* in the chromosomes. Subsequently, IS*Val1* was identified in the chromosome six genomes from 2015 and 2016. In the Huanghua data set, IS*Val1* was found in six genomes distributed in various locations of chromosomes. Remarkably, four and six insertion events of IS*Val1* were identified in chromosomes I and II of strain HB23, respectively. Further analysis showed that IS*Val1* insertion resulted in the disruption of the gene encoding lipid A biosynthesis lauroyl acyltransferase (Fig. S2) and ATP-dependent DNA helicase PcrA (Fig. S2) in chromosomes I and II, respectively. In addition, in strain HB23, a transposase belonging to the IS4 family was inserted between *fliD* and *flaA* within the gene cluster encoding flagellar biosynthesis.

### Pan-genomic analysis revealed the HGT of GIs assisted by IS.

**FIG 4:**
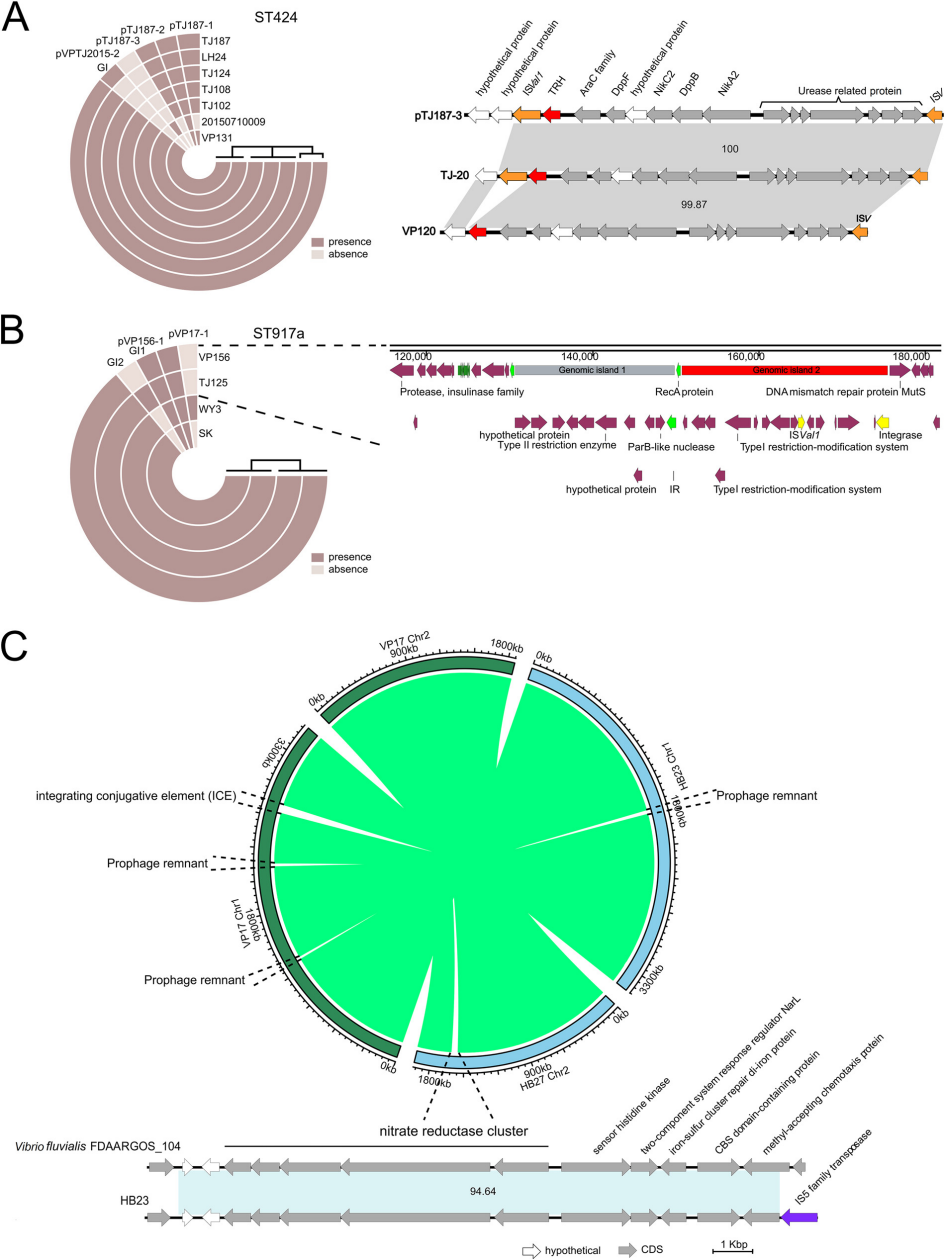
Horizontal transfer of a genomic island. (A) Horizontal transfer of a *trh*-*nik*-*ure* genomic island from the chromosome to the plasmid mediated by IS*Val1*. (B) Schematic diagram showing the comparative genome analysis of ST917a isolates and the integration of two genomic islands near *recA* in VP156 and TJ125. (C) Comparative genome analysis between the V. parahaemolyticus strain VP17 and HB23. Linear comparison of an inserted genomic island between V. parahaemolyticus genome HB23 and Vibrio fluvialis genome FDAARGOS_104 showing the acquisition of a nitrate reductase cluster.

As MLST revealed that three shrimp farms shared several common STs (e.g., ST424, ST1743, ST864, and ST415), subsequently, we conducted a pan-genomic analysis to identify potential HGT of MGEs. Interestingly, the formation of MGEs possibly associated with virulence and subsequent HGTs was observed in one ST1 and three ST917a and ST1800 isolates. In one sequenced ST1 strain, TJ-20, IS*Val1* was inserted into the flanking region next to the *trh*-*nik*-*ure* genomic island (Fig. 4A).

Another new MGE with IS*Val1* was found in ST917a strains. A genomic analysis of 2015 strain VP156 revealed that it harbors a 19.5-kb genomic island (GI-1) that disrupted the original *recA* gene with a 14-bp insert repeat. Eleven open reading frames (ORFs) were identified in this genomic island containing a second non*-*V. parahaemolyticus *recA* gene, which differed from the indigenous V. parahaemolyticus *recA* gene (87%/82% coverage/identity). In 2016, compared with strain VP156, strain TJ125 had another 24.3-kb genomic island (GI-2) next to GI-1, which has 16 ORFs, including one copy of IS*Val1* and an integrase gene (Fig. 4B). Interestingly, GI-2 was also found in ST150 strains VP17 and TJ107 but without IS*Val1*.

We further compared the genome structures between two ST150 strains. Results showed that strains VP17 and HB23 possess two and one unique prophage remnants, respectively (Fig. 4C). Additionally, strain VP17 processes an integrating conjugative element (ICE), which is widely found in Vibrio cholerae, Proteus mirabilis, *Shewanella* spp., and other species. Interestingly, strain HB23 harbored a 17.8-kb genomic island with 99% DNA similarity to a Vibrio fluvialis strain which encodes respiratory nitrate reductase, nitrate/nitrite transporter NarK, nitrate/nitrite sensor protein NarX, and nitrate/nitrite response regulator protein NarL (Fig. 4C). A denitrification assay showed that strain HB23 is able to grow in denitrification agar plates, which is not observed for another two ST150 isolates (Table S5). This observation suggested that the acquisition of this 17.8-kb genomic island might confer strain HB23 denitrification capacity under aerobic conditions.

10.1128/mSystems.01254-20.7 TABLE S4 Homology of integrating conjugative element identified in strain VP17. Download Table S4, XLSX file, 0.01 MB. Copyright © 2021 Fu et al. 2021 Fu et al. This content is distributed under the terms of the Creative Commons Attribution 4.0 International license.

10.1128/mSystems.01254-20.8 TABLE S5 Denitrification assay of ST150 strains. Download Table S5, XLSX file, 0.01 MB. Copyright © 2021 Fu et al. 2021 Fu et al. This content is distributed under the terms of the Creative Commons Attribution 4.0 International license.

In 2017, a 15-kb *trh*-*nik*-*ure* genomic island with IS*Val1* was also identified in the plasmid of an ST424 strain, TJ187 (Fig. S3), suggesting that the genomic island has horizontally transferred to ST424.

10.1128/mSystems.01254-20.3 FIG S3 Schematic diagrams of three plasmids identified in strain TJ187. Genes are denoted by arrows and colored based on gene function classification. The innermost two circles indicate the GC skew [(G – C)/(G + C)] and the GC content. The gene content of the plasmids is illustrated with DNAplotter. Download FIG S3, TIF file, 2.5 MB. Copyright © 2021 Fu et al. 2021 Fu et al. This content is distributed under the terms of the Creative Commons Attribution 4.0 International license.

To determine whether the *trh*-*nik*-*ure* genomic island was also presented in other STs, we searched public genomes and found that one Vibrio diabolicus strain and 27 V. parahaemolyticus strains were positive for this genomic island, which was located on both chromosome 1 and chromosome 2 (Table S6). These V. parahaemolyticus strains could be divided into 10 STs, including ST34, ST631, ST417, and ST36, the majority of which were found in clinical samples. These observations suggest that *trh*-*nik*-*ure* genomic islands were frequently transferred via plasmids.

10.1128/mSystems.01254-20.9 TABLE S6 Distribution of the *trh-nik-ure* genomic island in the public genomes of *Vibrio* spp. Download Table S6, XLSX file, 0.01 MB. Copyright © 2021 Fu et al. 2021 Fu et al. This content is distributed under the terms of the Creative Commons Attribution 4.0 International license.

### Plasmid profile of sequenced isolates.

The formation of *trh*-positive plasmids promoted us to take a survey of the plasmid profiles of all sequenced isolates to understand whether plasmids were horizontally transferred. For the isolates with multiple plasmids, Nanopore sequencing technology was employed to obtain the complete plasmid sequence. Comparative genome analysis of 60 sequenced strains from three shrimp farms also found that nine plasmids were variably presented, namely, pVP17-1 (70.1 kb), pVP17-2 (78.0 kb), pVP17-3 (68.7 kb), pTJ187-2 (71.7 kb), pTJ187-3 (86.5 kb), pVPTJ15-2 (83.3 kb), p 114-2 (36.9 kb), pLH80-1 (83.3 kb), and pVP156-1 (106.2 kb) (Table S7).

10.1128/mSystems.01254-20.10 TABLE S7 General information of plasmids identified in this study. Download Table S7, XLSX file, 0.01 MB. Copyright © 2021 Fu et al. 2021 Fu et al. This content is distributed under the terms of the Creative Commons Attribution 4.0 International license.

**FIG 5:**
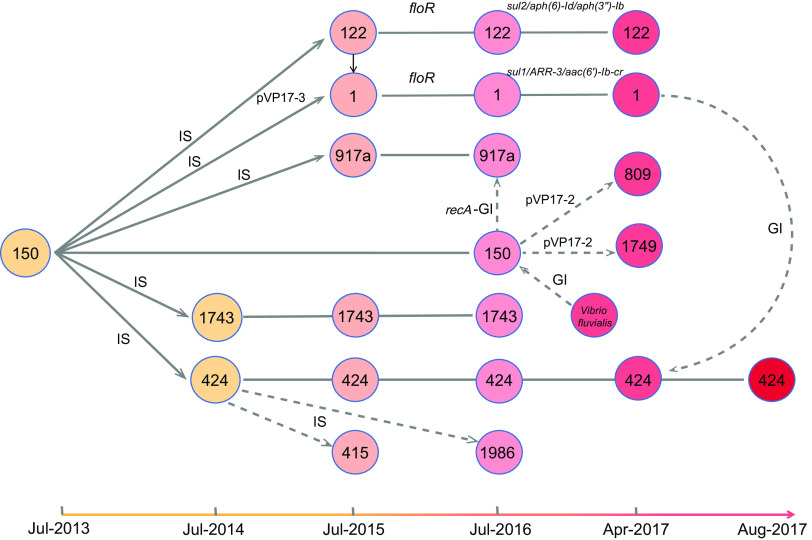
Schematic diagrams of evolutionary events observed in the Tianjin, China, shrimp farm. Horizontal gene transfer observed in the Tianjin shrimp farm from 2013 to 2017. The sequence types obtained from different years are indicated in the circles, which are placed in a sequential order. Horizontal transfer of plasmids, insertion sequences (IS), and genomic islands (GI) are indicated on the black lines connecting the circles. The dashed lines indicate speculative transfer routes.

Horizontal transfer of plasmids was also commonly observed among the sequenced genomes (Fig. 5). The *pirAB^vp^*-positive plasmid pVP17-1 was found in one ST150, three ST1743, five ST415, and four ST424 isolates (Table S1). The remaining five *pirAB^vp^*-negative plasmids were variably presented. pVP17-2, with 99% DNA similarity to p1937-2, was first found in ST150 and then identified in ST122, ST1749, and ST809. pVP17-3 (no significant hit with public genome data) was initially found in an ST150 strain but later was identified in ST1 strains in 2015. All of these results pointed to isolate VP17 as a potential genetic donor for the remaining isolates. The introduction of an ST150 strain might have led to the subsequent transfer of IS*Val1* and plasmids into the ST917a, ST1743, and ST424 in 2014 (Fig. 3).

ST917a isolate VP156 has a 106.2-kb plasmid with 95%/99% (coverage/similarity) to plasmid pVPE61b, which was found in all ST917a strains and some ST1743 strains. pTJ187-2 was exclusively found in ST424 strains which contain an f237 prophage harboring *zot* and *ace.* This prophage was also found in the chromosome of all ST424 strains, indicating prophage transfer between the plasmid and chromosome. pTJ187-3 was only identified in ST424 strains TJ102, TJ108, TJ124, TJ187, and LH24, of which strain TJ187 received the 15-kb PAIs.

## DISCUSSION

### IS facilitated the formation of resistance and pathogenicity islands (PAIs).

V. parahaemolyticus is one of the most significant bacterial pathogens, mostly because of its exceptional ability to adapt to marine environments and infectivity to humans and a wide range of marine animals, such as shrimp (22), fish, and mollusks (27). Previous studies suggested that the serotype O3:K6 clone acquired several genomic islands and subsequently caused the pandemic worldwide since 1996. Our previous study found that IS*Val1* can transfer between plasmids and chromosomes both *in vivo* and *in vitro* (22). In this study, we first observed the formation of resistant MGEs mediated by IS*Val1*, as a result of selection pressure from the use of antibiotics. As AHPND in shrimp spread, various antibiotics were used for treatment, which might activate the transposition of IS*Val1*. The stepwise acquisition of resistance MGEs in the plasmid of ST1 and ST122 strains mirrors the usage of antibiotics. Application of florfenicol in 2014 resulted in the independent acquisition of *floR*-carrying plasmids in 2015. Thereafter, subsequent application of enrofloxacin, streptomycin, and sulfamethoxazole resulted in the emergence of multidrug resistance (MDR) plasmids in 2016.

Interestingly, our data indicate that the simultaneous use of different classes of drugs has limited benefits due to the occurrence of the horizontal acquisition of MGEs, leading to acquisition of MDR in one event. The rapid and diverse acquisition events of resistance MGEs point out the potential of the environment as a reservoir for resistance genes. Thus, our results indicate that the simultaneous usage of over two antibiotics during the outbreak has, in fact, hastened the emergence of multidrug resistance (28).

Although no *in vitro* experimental evidence is presented in this study, the activation of MGEs by antibiotics at subinhibitory concentrations has been reported in several previous studies. For instance, Beaber et al. demonstrated that SXT elements from Vibrio cholerae are induced by antibiotics triggering the SOS response (29). Likewise, Scornec et al. found that subinhibitory concentrations of tetracycline activated the transfer of MGEs in Enterococcus faecalis through an antiattenuation mechanism (30).

Next, we looked for the other signatures of microevolution among the V. parahaemolyticus genomes mediated by insertion sequence IS*Val1*. A thorough search of the insertion sites of IS*Val1* in the 60 genomes identified 61 types of insertion sites, of which the *trh-nik-ure* genomic island flanked by IS*Val1* was found in both the chromosome of ST1 in 2016 and the plasmid of ST424 in 2017. blastn searches showed that the *trh* genes were flanked by different ISs in numerous V. parahaemolyticus genomes (Table S6), indicating the horizontal transferability of this genomic island. The genomic island containing the *tdh* or *trh* genes flanked by ISs in V. parahaemolyticus was found previously (26, 31). The HGT of this type of genomic island among different *Vibrio* spp. was subsequently found by Nishibuchi et al. (32). In their study, the *tdh* gene was transferred between different replicons by transposition, and plasmids could have been the vehicles for the *tdh* gene transfer between different organisms, suggesting the *tdh* gene was transferred first from the *tdh*-bearing non-*Vibrio* organisms to a *Vibrio* species. Likewise, Xu et al. found that one human-pathogenic variant (ST631) evolved independently through acquisition of distinct PAIs containing *trh*, suggesting that nonpathogenic V. parahaemolyticus harbored the regulatory mechanisms involved in managing newly acquired virulence elements (33). These studies suggested that the coordinated expression of *tdh/trh* is an ancient mechanism and that horizontally acquired genomic islands can incorporate into existing regulatory networks with sufficient expression (33).

Acquisition of PAIs or fitness islands via HGT provides an essential mechanism for the bacterial adaptation to different niches. Another interesting horizontal transfer event is the disruption of *recA* by a genomic island in an ST917a strain followed by the second insertion from a second genomic island, which might come from ST150 strains. The potential roles of two genomic islands will be examined in a future study. However, it has been shown that the inserted *recA* could protect against DNA damage induced by UV irradiation in V. cholerae (34). In another study, Chen et al. also identified an O4:KUT-recAin type strain with an insertion of a genomic island in *recA* alleles, which has become a new emerging serotype since 2014 and exhibited strong acid resistance at pH 4.9 (35). Additionally, strain HB23, obtained from the Huanghua farm, acquired a fitness island, which confers to it the denitrifying capacity under aerobic conditions. These observations suggested that genomic surveillance at the farm would facilitate the early warning about the new virulent genotype.

### Real-time genomic surveillance of pathogens provided important insights into the prediction of emergent populations.

In this study, we selected three shrimp farms to observe the *in vivo* evolution of V. parahaemolyticus. Despite the three farms exhibiting distinct V. parahaemolyticus populations and the surveillance periods varying from 6 to 8 years, the three data sets exhibited similar evolutionary trajectories—frequent transposition activities resulting in the HGT of virulence or fitness islands.

**FIG 6:**
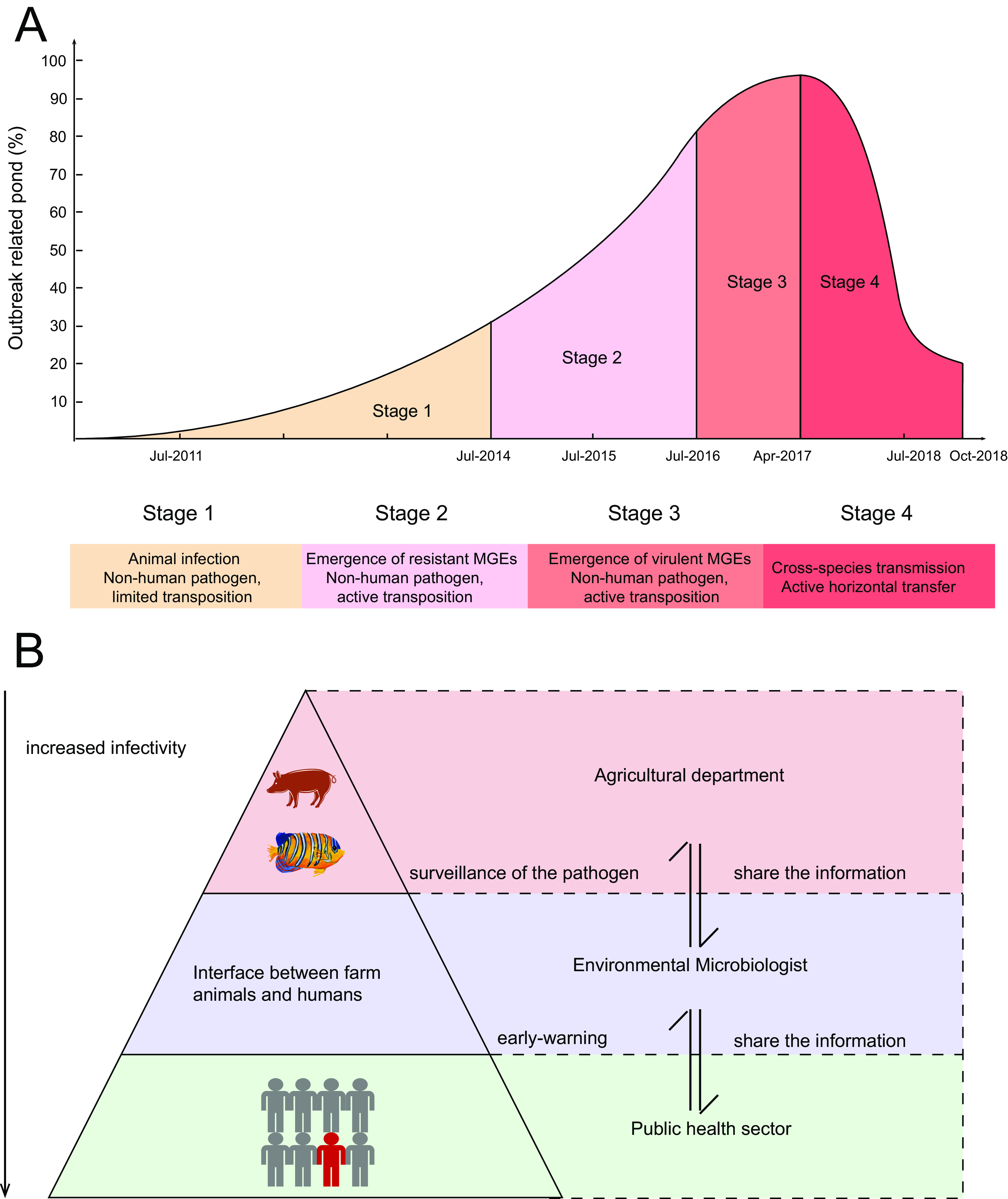
(A and B) Four milestone evolutionary stages of bacterial pathogens (A) and suggested prevention strategies (B). Evolutionary trajectories were divided into the following four stages: stage 1, transfer of IS*Val1* from plasmid to chromosome; stage 2, formation of resistant MGEs; stage 3, formation of virulent MGEs; stage 4, horizontal transfer of MGEs and plasmid. The decline of the outbreak-related pond is due to the discarding of the pond.

Thus, we summarized that there were four stages of the microevolution of V. parahaemolyticus (Fig. 6A). In stage I (from 2011 to 2014), occasional outbreaks occurred on the shrimp farm accompanied with the transfer of IS*Val1* from plasmid to chromosome. Then AHPND outbreaks spread into more ponds, resulting in wide use of antibiotics. In stage II (2015), antibiotic usage facilitated the formation of resistant MGEs mediated by insertion sequences. Resistant strains started to emerge with resistant MGEs thanks to IS*Val1*. In stage III (2016), IS*Val1* was inserted into the chromosomes of more STs, resulting in the formation of virulent MGEs. One IS*Val1* was inserted into the flanking regions of *trh*. Meanwhile, another genomic island might horizontally transfer from ST150 to ST917a, conferring on them a stronger resistance to UV treatment. In stage IV (2017), horizontal transfers of GIs associated with pathogenicity and fitness were found in ST424, ST917a, and ST150 strains.

The HGT of pathogenicity islands can be suggested as a signal of early warning for the emergence of virulent populations. Therefore, various prevention measures can be adopted at the different stages to avoid the epidemic deterioration. For instance, at stage III, as antibiotics trigger the transposition activity of resistance islands, antibiotics should be used with caution. At stage IV, critical measures should be adopted to prevent the spillover of the pathogen. Subsequently, we conducted 6-month genomic surveillance on another shrimp farm, which prevented the spread of the outbreaks and controlled the formation of virulence MGEs.

Continuous pathogen surveillance is necessary to understand the health risks of a certain pathogen and to help predict future evolutionary consequences, which serves as the basis for an intermediate intervention. Our recent genomic surveillance at a chicken farm identified an anthrax toxin-positive Bacillus cereus strain in a probiotic product used as a feed supplement, which was transferred into the groundwater and to a nearby fish farm (36). Continuous surveillance of the groundwater subsequently monitored the contamination of groundwater and led to the early prevention of a potential epidemic (36).

HGT has had a major role in the acquisition of genes associated with virulence in Vibrio vulnificus (37). Our study showed that the activity of HGT mediated by IS could be used as an indicator to predict potential human pathogens. The emergence of a recent Shiga toxin-producing E. coli O104:H4 clone in Germany was another example caused by horizontal transfer of a toxin-producing phage into an enteroaggregative E. coli strain (12).

Our results also suggest that clinical surveillance is not sufficient to warn about an oncoming epidemic at an early stage. Three levels of surveillance program to monitor, respectively, the animal infection at the farm or in wildlife, prepandemic transmission at the interface between animals and humans, and sporadic infection at sentinel hospitals should be set up (Fig. 6B). Information should also be shared between agriculture and public health sectors to address the emerging epidemic in a timely manner. Further studies should examine the possibility of real-time metagenomic surveillance for all pathogens. Efficient monitoring of the interfaces between the environment, wildlife and farm animals, and humans may contribute to reducing the dissemination of pathogens and ultimately to detect early signals of an epidemic.

### Conclusion.

Real-time genomic surveillance of V. parahaemolyticus in a shrimp-farming region found that IS*Val1* mediated the HGT of prophages, plasmids, and genomic islands among V. parahaemolyticus in response to adverse external environmental and selection pressure. Analysis of evolutionary events in this study has led to a better understanding of the underlying mechanisms of pathogen emergence. It is a matter of urgency that we need to strength the surveillance of zoonotic infectious agents at the interfaces between wildlife, farm animals, the environment, and humans.

## MATERIALS AND METHODS

### Sample collection, bacterium isolation, and identification in shrimp farms.

From 2011 to 2018, 60 shrimp were collected in July or April annually from three shrimp-farming regions near Tianjin (from 2011 to 2018), Tangshan (from 2011 to 2017), and Huanghua City (from 2011 to 2016), China (Fig. S1). Hepatopancreas from shrimp were aseptically disaggregated in 100 ml alkaline peptone water and streaked on thiosulfate citrate bile salts sucrose (TCBS) plates at 28°C for 24 h. The bacterial identification of suspected colonies obtained from TCBS was conducted as described previously (22).

MLST was conducted as described by Gonzalez-Escalona et al. (23). PCR detection of the virulence genes *tdh*, *trh*, *pirAB^vp^*, and IS*Val1* was conducted as previously described (24, 25). The presence of T3SS1 and T3SS2 was verified by PCR of the genes *vcrD1* and *vscC2*, respectively (26).

### Whole-genome sequencing, *de novo* assembly, and annotation.

Total DNA was extracted from overnight culture of the isolates with the Wizard Genomic DNA kit (Promega, Madison, WI, USA) for whole-genome sequencing. High-throughput genome sequencing was carried out on Illumina platforms or a Nanopore platform at Novogene (Tianjin, China). The draft genome was assembled *de novo* with SPAdes version 3.0 (38). RAST was used to annotate the sequences of each genome determined with next-generation sequencing (39). PIRATE was used to build a comprehensive pan-genome of the population and identify accessory genes (40). Outputs of the pan-genome were analyzed with R version 3.2.3 (41).

### Identification of SNPs and phylogenetic analyses.

The core genome of V. parahaemolyticus defined by Gonzalez-Escalona et al. was used as the reference genome to call the single-nucleotide polymorphisms (SNPs) for V. parahaemolyticus (42). SNP calling was performed with a previously developed pipeline to guarantee that only genuine SNPs were included in the analysis (43).

RAxML version 7.8.6 was used with the generalized time-reversible model and a Gamma distribution to model site-specific rate variation (the GTR+⌈ substitution model; GTRGAMMA in RAxML) (44). Support for the ML phylogeny was assessed by 100 bootstrap pseudoanalyses of the alignment data, and the final tree was visualized in FigTree version 1.4.2 (http://tree.bio.ed.ac.uk/software/figtree/).

### Denitrification assay.

A denitrification medium (DM) was used to confirm the denitrification capacity of test strains under aerobic conditions. The composition of the DM was described previously (45). Strains HB23, TJ107, and VP17 were suspended in 0.9% saline solution and streaked onto the DM agar plates (with g · liter^−1^ NO_3_^−^-N 0.72). A Luria-Bertani (LB) medium was also inoculated as the control. The aerobic denitrification ability was assessed after incubation at 28°C for 48 h. All experiments were carried out in triplicate.

### Data availability.

The raw sequencing data were submitted to GenBank (NCBI) under the BioProject no. PRJNA503785.
