# Supplementary figures and images for: Continuous Genomic Surveillance Monitored the In Vivo Evolutionary Trajectories of Vibrio parahaemolyticus and Identified a New Virulent Genotype
Source: mSystems. 2021 Jan 19;6(1):e01254-20. doi: 10.1128/mSystems.01254-20 (PMC7820670; doi:10.1128/mSystems.01254-20)

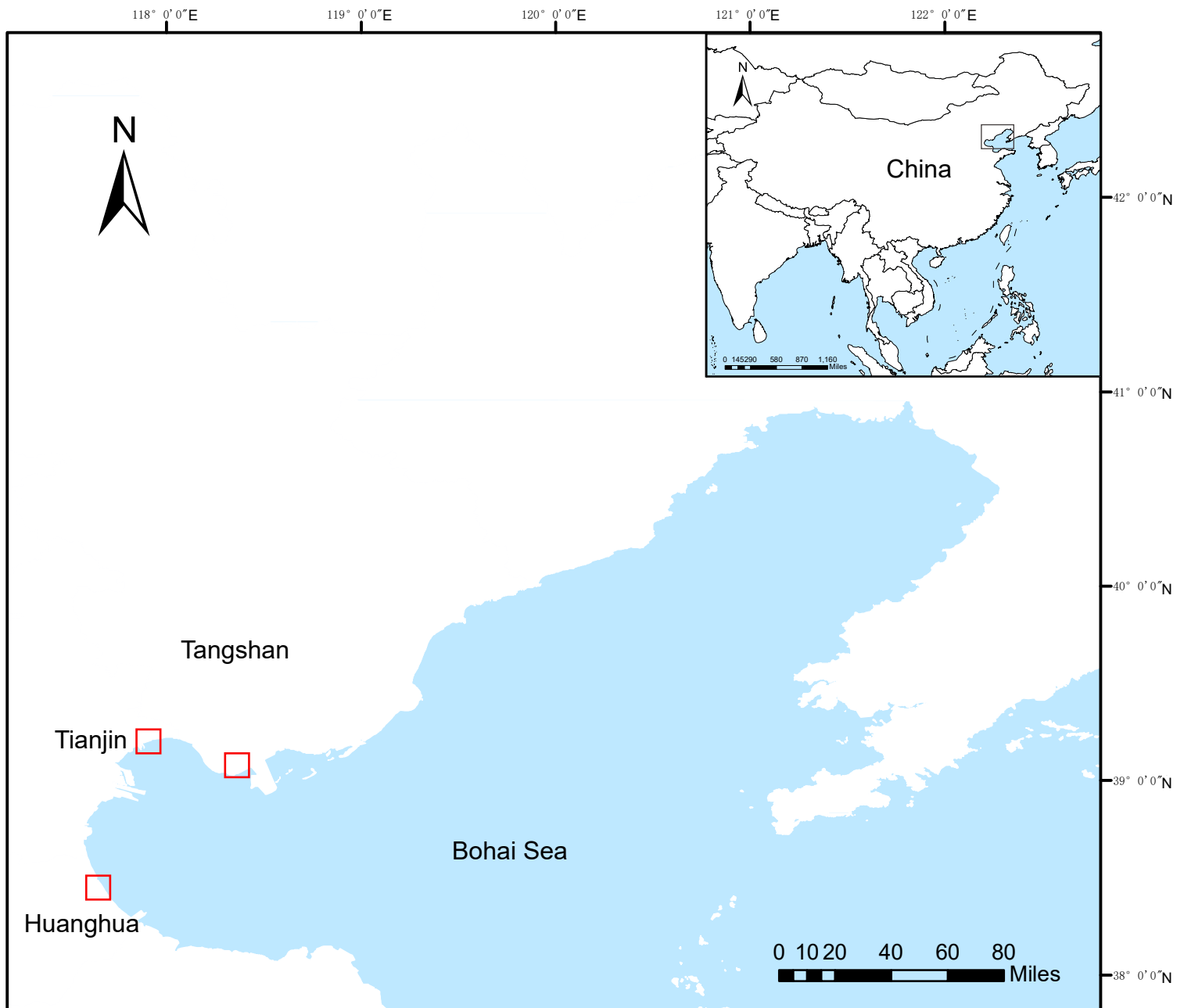

Supplement: FIG S1 [file mSystems.01254-20-sf001.pdf]

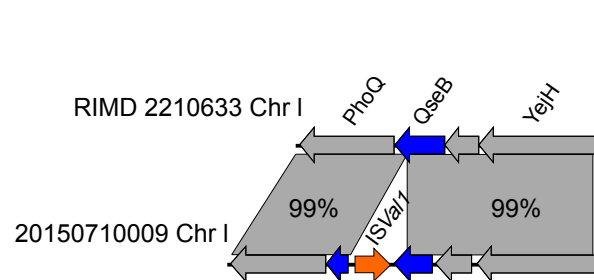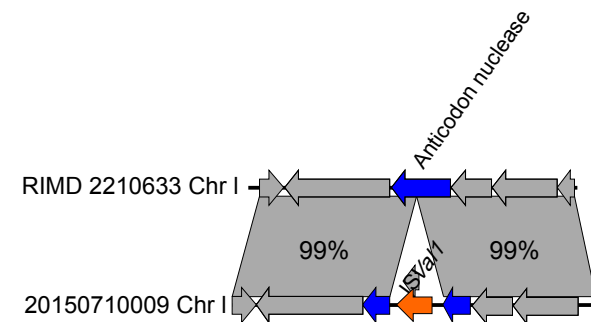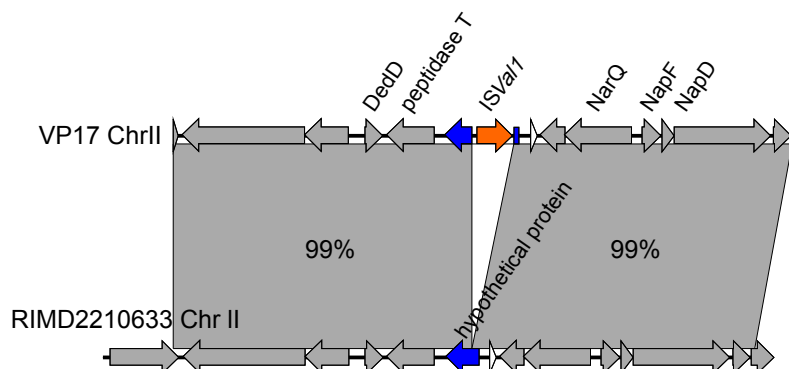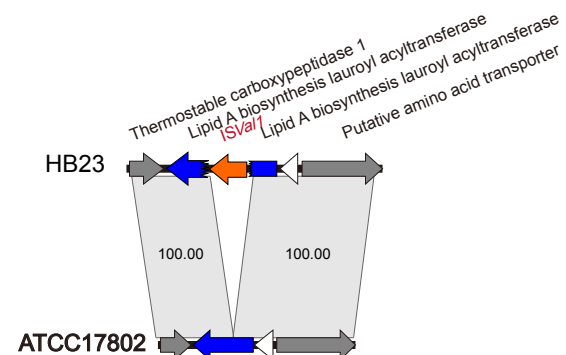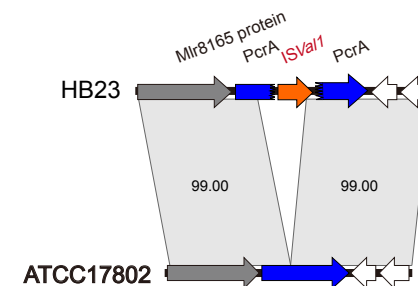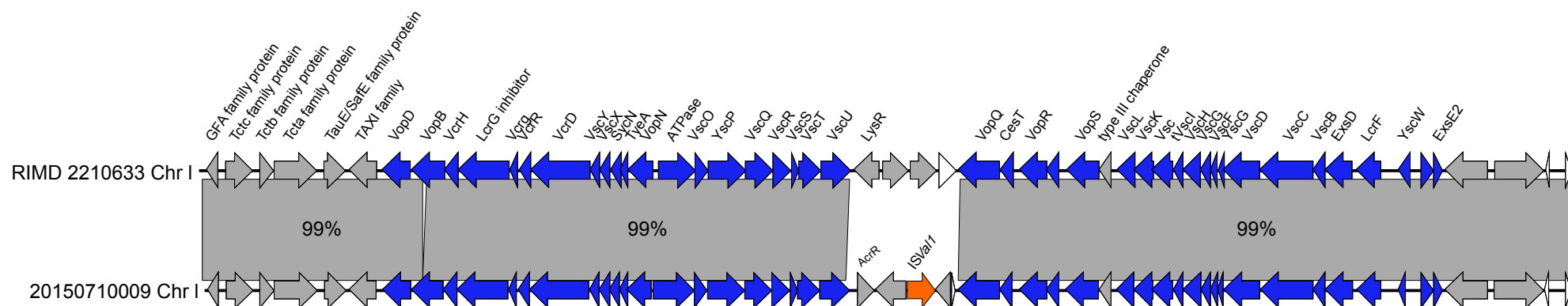

Supplement: FIG S2 [file mSystems.01254-20-sf002.pdf]

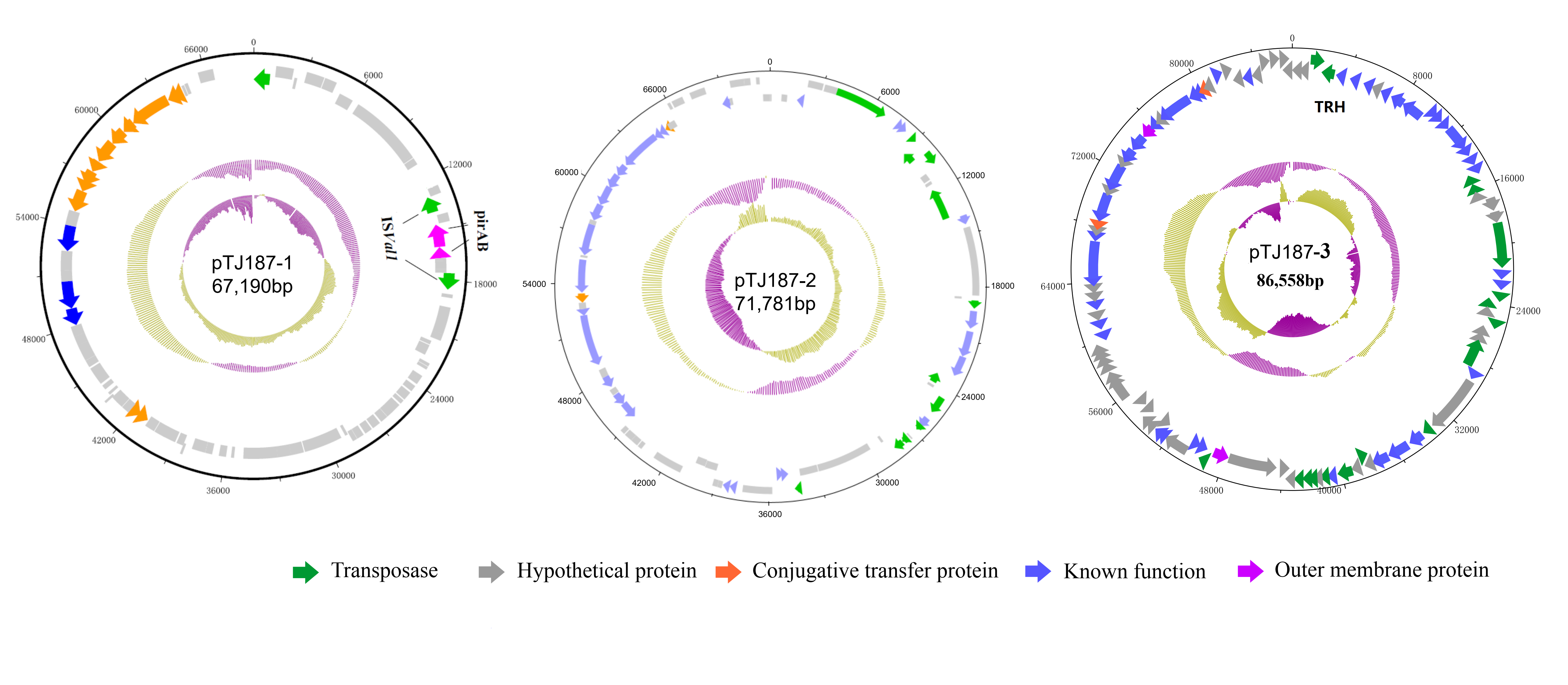

Supplement: FIG S3 [file mSystems.01254-20-sf003.tif]
